# Annual Glyphosate Treatments Alter Growth of Unaffected Bentgrass (*Agrostis*) Weeds and Plant Community Composition

**DOI:** 10.1371/journal.pone.0050643

**Published:** 2012-12-04

**Authors:** Collin W. Ahrens, Carol A. Auer

**Affiliations:** Agricultural Biotechnology Lab, Department of Plant Science, University of Connecticut, Storrs, Connecticut, United States of America; Centro de Investigación y de Estudios Avanzados del IPN, Mexico

## Abstract

Herbicide resistance is becoming more common in weed ecotypes and crop species including turfgrasses, but current gaps in knowledge limit predictive ecological risk assessments and risk management plans. This project examined the effect of annual glyphosate applications on the vegetative growth and reproductive potential of two weedy bentgrasses, creeping bentgrass (CB) and redtop (RT), where the glyphosate resistance (GR) trait was mimicked by covering the bentgrass plants during glyphosate application. Five field plots were studied in habitats commonly inhabited by weedy bentgrasses including an agricultural hayfield, natural meadow, and wasteland. Results showed that annual glyphosate treatment improved bentgrass survivorship, vegetative growth, and reproductive potential compared with bentgrass in unsprayed subplots. In the second year of growth, RT plants had an 86-fold increase in flower number in glyphosate-treated subplots versus controls, while CB plants had a 20-fold increase. At the end of the three year study, plant community composition had changed in glyphosate-treated subplots in hayfield and meadow plots compared to controls. Soils in subplots receiving glyphosate had higher nitrate concentrations than controls. This is the first study to mimic the GR trait in bentgrass plants with the goal of quantifying bentgrass response to glyphosate selection pressure and understanding the impacts on surrounding plant communities.

## Introduction

Herbicide resistance has increased in weed species concurrent with the expanding acreage used for herbicide resistant crops in the U.S., but the long-term environmental impact of these traits on plant communities is largely unknown [1]. Crops and weeds have become resistant to the herbicide glyphosate [*N*(phosphonomethyl)glycine] through two mechanisms: 1) genetic engineering with the 5-enolpyruvylshikimate-3-phosphate synthase (EPSPS) transgene or glyphosate oxidoreductase (GOX) transgene, or 2) repeated glyphosate selection pressure coupled with physiological and genetic changes [2], [3], [4]. Genetic engineering has been used in the U.S. to commercialize glyphosate resistance (GR) in important agronomic crops (e.g. maize, cotton, soybeans, alfalfa, canola, and sugarbeet) and the trait has been found in feral GR crops (e.g. canola, volunteer corn) [5], [4]. Genetic engineering has been used to add the GR trait to two perennial turfgrasses, creeping bentgrass (*Agrostis stolonifera*) and Kentucky bluegrass (*Poa pratensis* L.). While GR creeping bentgrass remains under regulatory review in the U.S., the USDA recently declined to regulate GR Kentucky bluegrass because it was generated without using any DNA sequences from an organism deemed a plant pest [6], [7].

Unfortunately, the presence of the GR trait in diverse weed species is negatively affecting the utility of GR crops [8], [9]. Selection pressure has created GR and glyphosate tolerant ecotypes in diverse weed species such as Canadian horseweed (*Conyza canadensis*), carelessweed (*Amaranthus palmeri*), ryegrass (*Lolium* species), Indian goosegrass (*Eleusine indica*), annual ragweed (*Ambrosia artemisiifolia*), Johnsongrass (*Sorghum halapense*), Mexican fireplant (*Euphorbia heterophylla*) and narrowleaf plantain (*Plantago lanceolata*) [10], [1], [4]. The mechanism for GR in weeds appears to vary with evidence for EPSPS gene amplification, EPSPS gene mutation, vacuolar sequestration and other mechanisms [11], [12], [13]. Regardless of the mechanism, glyphosate selection pressure will cause the heritable GR trait to spread in weed populations via pollen-mediated gene flow, seed dispersal, and vegetative propagules. Without selection pressure, the GR trait will be governed by genetic drift and gene flow. However, a study of field mustard (*Brassica rapa*) showed that the GR trait can persist without selection pressure [14].

Plant populations and community assemblages are affected by natural forces such as plant competition, facilitation, and stochastic processes [15]. In addition, many plant communities are also intentionally or unintentionally impacted by management regimes including herbicides and herbicide drift. Glyphosate usage has increased dramatically in the U.S., and recent experiments have shown that glyphosate can be detected in some air and surface water samples [16], [17]. Deposition of sub-lethal glyphosate concentrations (drift) can negatively affect plant reproductive fitness [5]. However, there is no clear consensus about the effects of glyphosate on plant community assemblages or biodiversity. While some studies have reported that glyphosate applications had little impact on species richness or diversity in plant communities [18], other studies have reported significant changes [19], [20]. A U.K. study by Heard et al. [21] showed that herbicides reduced weed abundance in GR beet (*Beta*) and canola (*Brassica*) fields, but weed species diversity and species richness within the fields was affected.

**Figure 1:**
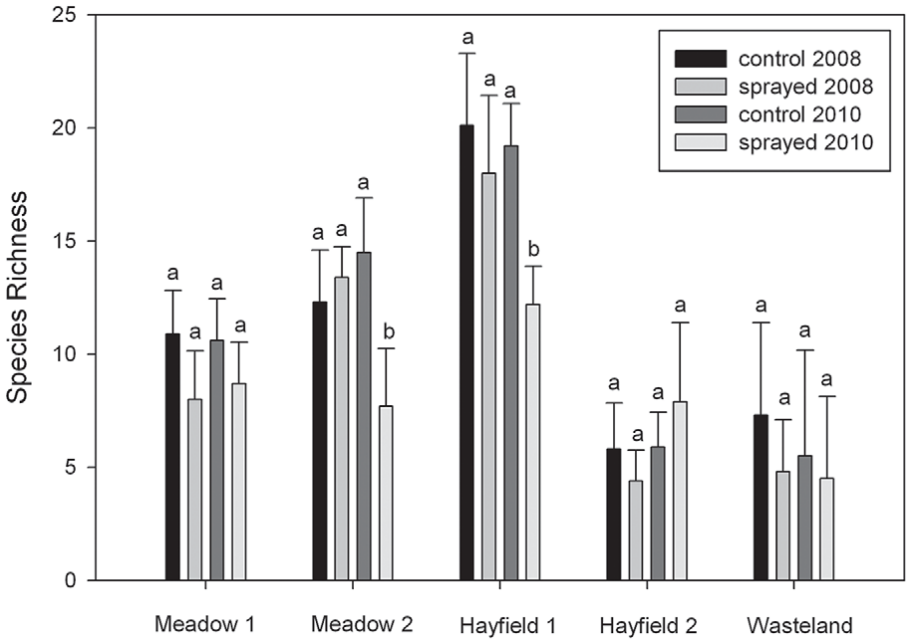
Species richness in subplots before the study (2008) and at the end of the experiment (2010). Error bars indicate SD and letters indicate differences within plot locations according to Fisher’s LSD test (α<0.05).

Risk assessments for the ecological impact of herbicide resistance trait require knowledge about host plant biology, crop-to-wild gene flow, and the ecology of the site for environmental release [22], [23], [24]. GR creeping bentgrass (*Agrostis stolonifera* L., CB) has generated concern because it is a cold-hardy perennial, a weed in many natural and managed habitats, part of a complex of closely-related species that hybridize through pollen-mediated gene flow, and able to spread vegetatively [25], [26], [27]. *Agrostis* species (bentgrasses) are common in early to mid-successional grasslands [28], implying that they readily establish in habitats with a variety of plant competion levels [29]. CB is a non-native species in the U.S. and a common weed outside of cultivation [30], [31], [32], [33]. Our previous research showed that that CB is common and abundant as a weed in various managed and disturbed habitats (e.g. roadsides, wastelands) in two ecoregions in the Northeastern U.S. [31]. Furthermore, CB frequently co-occurs with a closely-related, non-native *Agrostis* called redtop (*Agrostis gigantea* Roth., RT). This co-occurrence suggests a potential for pollen-mediated gene flow [31]. In another study, CB, four other bentgrass species, and interspecific CB hybrids were able to establish and compete in residential and roadside habitats [34]. CB has also been shown to persist in sand dune, salt marsh, meadow and polder habitats [35]. In the Western U.S., pollen-mediated transgene flow between CB and RT populations moved the GR trait long distances [36], and removal of the escaped GR CB plants has been problematic along irrigation ditches because glyphosate is the only herbicide approved for these waterways [37]. Thus, the complex of native and non-native *Agrostis* species already present in the U.S. support intraspecific and interspecific gene flow, although the temporal and spatial scales have not been defined [38], [37], [27], [39].

**Figure 2:**
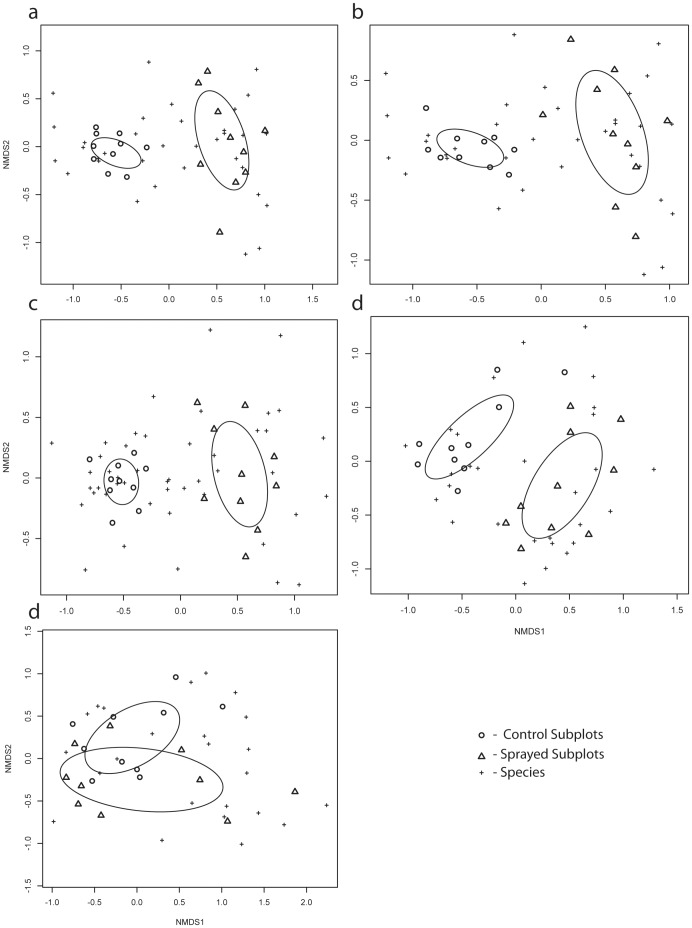
Non-multidimensional scaling of each plot using Bray-Curtis Dissimilarity. a) meadow 1, b) meadow 2, c) hayfield 1, d) hayfield 2, and e) wasteland. The ellipses enclose the 99% confidence intervals.

It has been reported that GR CB plants were not significantly different to their non-transformed counterparts with regard to infloresence development, reproductive morphology, pollen longevity, seed-set capacity or growth and establishment [40], [41]. However, these studies did not examine CB growth under glyphosate selection pressure which would reduce competition between CB and other plants in natural, cultural or agricultural landscapes. It is important to understand GR CB behavior with glyphosate selection pressure in order to predict its behavior as a weed, create predictive models of gene flow and dispersal, and quantify impacts on plant community assemblages. Therefore, the primary goals of this project were to: 1) examine the impact of annual glyphosate applications on established plant communities in agricultural and natural areas, and 2) determine if one glyphosate application per year could alter the survivorship, growth, and reproductive capacity of introduced CB and RT plants. In this project, the GR trait in the bentgrass plants had to be mimicked due to a variety of factors including the potential for transgene escape during flowering and seed production. Our first hypothesis was that plant community composition would be altered in subplots receiving glyphosate compared to control (unsprayed) subplots. Second, we predicted that CB and RT plants with mimicked GR in glyphosate-treated subplots would have increased growth and reproductive potential compared to control subplots. To our knowledge, this is the first study to examine the effects of glyphosate on weedy bentgrass plants in the context of natural and agricultural plant communities.

**Table 1:** Soil nutrients and pH in five field plots prior to the experiment.

Plot Name	Ca	Mg	P	K	NO_3_	pH
	kg/ha				mg/kg	
Meadow 1	3226^a^	433^a^	1^b^	164^b^	3.49	5.78
Meadow 2	2839^a^	519^a^	1^b^	147^b^	4.34	5.64
Hayfield 1	1101^b^	147^b^	0^b^	160^b^	1.26	5.82
Hayfield 2	1572^b^	283^a^	2^b^	247^a^	4.11	6.07
Wasteland	1466^b^	322^a^	44^a^	276^a^	3.92	5.76

Letters indicate significant differences within a column according to Fisher’s LSD test (α<0.05). No differences were observed for nitrate-N or pH.

## Materials and Methods

### Field Plots and Glyphosate Treatment

Five field plots (12 m×15 m) were established in Mansfield, Connecticut in the Lower New England Ecoregion, subecoregion Southern New England Coastal Hills and Plains. The plot names and locations were: Meadow 1, 41 49′ 30.30′′ N, 72 14′ 12.91′′ W; Meadow 2, 41 49′ 30.71′′ N, 72 14′ 18.43′′ W; Hayfield 1, 41 46′ 58.30′′ N, 72 13′ 07.59′′ W; Hayfield 2, 41 46′ 55.80′′ N, 72 13′ 10.56′′ W; and Wasteland, 41 49′ 01.25′′ N, 72 13′ 10.56′′ W. Meadow 1 and 2 were established in a natural meadow along the Fenton River. Hayfield 1 and 2 were placed on the upper and lower edges of an agricultural hayfield. The Wasteland plot was established in an area bordered by a road, cow pasture and wetlands, and dominated by the invasive grass canary reedgrass (*Phalaris arundinaceae* L.). Plots were not fertilized, irrigated or mowed during the 3 years of the study, and there was no known history of herbicide use at these sites. The overall intent was to mimic the localized application of glyphosate to control invasive plants or serious weeds in conservation areas, hayfields, or roadside wastelands. No permits were required for the project. Glyphosate was applied according to label specifications using appropriate personal protective equipment. All field plots were located on property owned by the University of Connecticut and did not contain any species of special concern.

**Table 2:** Mean bentgrass biomass (g dry weight) and survivorship (percent) for introduced creeping bentgrass (CB) and redtop (RT) plants at the end of the field experiment (2010).

Species	Treatment	Biomass	Meadow 1	Meadow 2	Hayfield 1	Hayfield 2	Wasteland	Total
CB	sprayed	5.85^a^ (7.85)	100	60	80	40	0	56
	control	0.0^b^ (0)	0	0	0	0	0	0
RT	sprayed	25.30^a^ (22.13)	100	20	60	60	40	56
	control	0.1^b^ (0)	0	0	20	0	0	4

Standard deviation is given in parentheses for plant biomass and letters indicate differences within bentgrass species according to Fisher’s LSD test (α<0.05).

A 2×2 factorial experiment was set up in each plot using a randomized complete block design with two management regimes (annual glyphosate spray or non-sprayed control) and two weedy, non-native bentgrass species (CB or RT). Each treatment group in the factorial design was replicated five times creating 20 randomized subplots with a size of 3 m^2^. Soil samples (combination of two soil cores 2.2 cm×10.2 cm depth) were taken in each subplot prior to the study and 3 years later at the same time of year (Oct.-Nov.). Samples were analyzed for pH and nutrients using a modified Morgan test and a soil test for nitrates (University of Connecticut Soils Testing Lab, Storrs, CT). The soil texture for all plots was either sandy loam or loamy sand. Weather data were obtained from the National Oceanic and Atmospheric Administration (NOAA, (www.noaa.gov)). Yearly rainfall totals from November 1 to October 31 were: 2008, 375 cm; 2009, 310 cm; 2010, 304 cm. Mid-summer rainfall was: June, 2008, 10.8 cm; July, 2008, 11.5 cm; June, 2009, 14.5 cm; July, 2009, 19.5 cm; June, 2010, 11.1 cm; July, 2010, 8.1 cm. Mid-summer mean maximum temperatures (C°) were: June, 2008, 24.2; July, 2008, 26.4; June, 2009, 21.1; July, 2009, 24.1; June, 2010, 24.8; July, 2010, 27.8.

**Figure 3:**
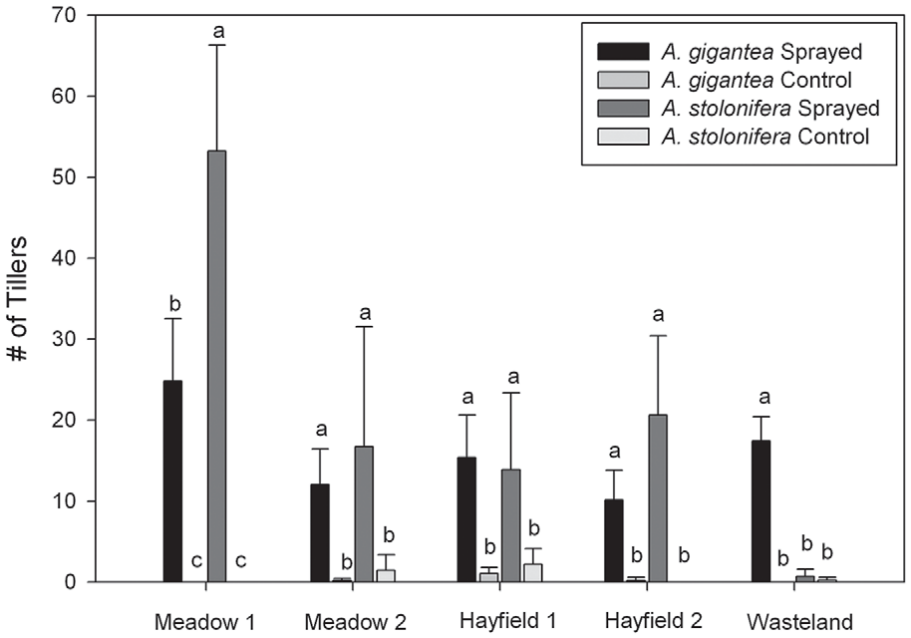
Number of tillers on creeping bentgrass (CB) and redtop (RT) plants in the last year of the field study (May, 2010). Error bars indicate SD and letters indicate differences within plot location at according to Fisher’s LSD test (α<0.05).

In 2007, conventional (non-genetically engineered) CB ‘Penn A-4’ and RT ‘unspecified’ (Des Moines Forage and Turf Seed Corporation, Ankeny, IA) plants were grown at the university research farm with biweekly mowing (1.27 cm height) and two fertilization treatments at a rate of 0.45 kg per 0.0093 ha for N, P and K (19-19-19, Lesco fertilizer, Troy, MI). Genetically engineered GR CB were not used in the study because: 1) we did not have permission to use commercially-developed, patented, transgenic GR CB seeds, 2) the U.S. government would be unlikely to provide permits for experimental field trials since the bentgrass plants needed to flower, release pollen, and produce seed in order to measure reproductive potential, and 3) to our knowledge, RT has not been engineered to contain the GR trait.

**Table 3:** Mean number of flowers produced by *A. gigantea* (RT) and *A. stolonifera* (CB) plants in glyphosate-treated subplots or unsprayed controls.

Species	Treatment	2008	2009	2010
**RT**	Control	0	213^b^ (92)	0^b^ (0)
	Glyphosate	0	18,377^a^ (16,032)	7959^a^ (8551)
**CB**	Control	0	104^b^ (46)	0^b^ (0)
	Glyphosate	0	2,042^a^ (1310)	1346^a^ (789)

Standard deviation is given in parentheses and letters indicate differences within bentgrass species and year according to Fisher’s LSD test (α<0.05).

In May, 2008, small plugs (3 cm×3 cm) of acclimated CB or RT were transplanted into the center of 100 subplots (20 subplots×5 plots). Measurements of bentgrass growth were taken monthly (May-October) for three years including: surface area covered, maximum leaf height, number of tillers (stolons), number of panicles, and number of flowers. All vascular plant species in each subplot were identified and monitored during the three year study. At the end of the third year (November, 2010), bentgrass survival and above-ground plant biomass were recorded. Subplots with CB or RT plants were randomly assigned to two treatment groups: annual glyphosate treatment or control (no herbicide). Subplots receiving glyphosate (1.69 kg per hectare, Roundup Pro ®, Monsanto Company, St. Louis, MO) were sprayed once per year on June 24–26^th^ using a hand-held wand with a single boom Teejet XR8004VS nozzle (Wheaton, IL) and a backpack sprayer (SP system backpack, Santa Monica, CA). *Agrostis* plants within the sprayed plots were temporarily covered with white plastic bags (0.5 mm thickness) for 10–15 minutes to mimic the herbicide resistance trait. Control subplots received no weed management during the experiment.

At the end of the experiment, dependent variables were examined using analyses of variance (SAS ver. 9). Soil nutrient levels were analyzed as possible covariates. Data from all subplots were combined to analyze treatment, year (treated as a random variable) and bentgrass species interaction effects. Survivorship was analyzed using a probit model to determine the significance of glyphosate application. Probit analysis was chosen because this type of regression analysis was developed for biological systems involving quantal responses (e.g. death) to differing dosages of a toxin or a stress (e.g. glyphosate application) [42], [43].

To assess changes in plant community composition, subplots were graphed on a two-axis graph using non-multidimensional scaling. The Bray-Curtis Dissimilarity between each subplot was calculated and the layout was based on the lowest amount of stress between actual dissimilarity and graphed dissimilarity. Confidence intervals (99%) were calculated and drawn for both control and sprayed subplot groups within each plot. If the confidence intervals did not overlap, then the communities in the subplot treatments (control vs. sprayed) were considered to be different [44].

## Results

### Effects of Glyphosate on Plant Community Composition and Soil Nitrogen

The five plot sites were typical examples of a natural meadow, hayfield or wasteland plant community that frequently contain non-native bentgrasses in our ecoregion. The high plant community diversity in Hayfield 1 could be attributed to cultivation of agronomic species such as smooth brome (*Bromus inermis* Leyss.) and timothy (*Phleum pratense* L.), plus the naturalization of native and non-native species such as RT, CB, velvet bentgrass (*A. canina* L.), colonial bentgrass (*A. capillaris* L.), rough bentgrass (*A. scabra* Willd.), rough goldenrod (*Solidago rugosa* Mill.), white panical aster (*Symphyotrichum lanceolatum* (Willd.) G.L. Nesom), poverty oatgrass (*Danthonia spicata* (L.) P. Beauv. Ex Roem. & Schult.), Kentucky bluegrass, fowl bluegrass (*Poa palustris* L.) and annual bluegrass (*Poa annua* L.). The Hayfield 2 plot had a lower species richness value because it contained mostly cultivated species including smooth brome and oat (*Avena sativa* L.). Meadow 1 was dominated by coastal plain joe pye weed (*Eupatoriadelphus dubius* (Wild. Ex Poir.) King & H. Rob.), rough goldenrod, giant goldenrod (*Solidago gigantea* Aiton), flat top goldenrod (*Euthamia graminifolia* (L.) Nutt.) and arrowleaf tearthumb (*Polygonum sagittatum* L.). Meadow 2 contained plants such as black raspberry (*Rubus occidentalis* L.), American red raspberry (*Rubus idaeus* L.), flat top goldenrod and rough goldenrod. The low species richness in the Wasteland plot was due to the predominance of reed canarygrass, although chicory (*Cichorium intybus*), Canada thistle (*Cirsium arvense*), bull thistle (*Cirsium vulgare*), Kentucky bluegrass and prickly lettuce (*Lactuca serriola*) were also identified.

After three years in the study, the glyphosate-treated subplots in Meadow 2 and Hayfield 1 had lower plant species richness (14.5 to 7.7 species per subplot, and 19.2 to 12.2 species, respectively) and altered plant community assemblages compared to control subplots (Fig. 1). These two plots also had the highest number of species prior to the study (*P*<0.0001). This result contrasts with the meta-analysis performed by Sullivan and Sullivan [18] which found that glyphosate did not change species richness. However, while glyphosate treatment in Meadow 1, Hayfield 2, and Wasteland subplots did not alter the metric of plant species richness, changes in plant community assemblages were observed. Plant community composition shifted in four plot sites due to glyphosate treatment (Fig. 2). This change could largely be attributed to the increased occurrence of early and late annuals such as fall panicum (*Panicum dichotomiflorum* Michx.), witchgrass (*Panicum capillare* L.), large crabgrass (*Digitaria sanguinalis* (L.) Scop.), Virginia pepperweed (*Lepidium virginicum* L.) and yellow rocket (*Barbarea vulgaris* W.T. Aiton) in sprayed subplots. The Wasteland plot was an exception to this trend (Fig. 2) due to the localized dominance of the invasive reed canarygrass, which quickly re-established itself after glyphosate treatment.

Soil samples taken before the experiment showed differences in soil fertility between plots (Table 1). The high concentration of P and K in the Wasteland plot was most likely due to previous land use for crop production, manure storage and/or manure application (personnal communication, Tom Morris). Meadow plots 1 and 2 had higher calcium concentrations than other plots, and Hayfield 1 had a lower Mg level (Table 1). However, analysis of covariance indicated that the differences in soil Mg, Ca, P, and K did not affect bentgrass growth or survivorship. At the end of the third year, soils in subplots receiving glyphosate had higher nitrate concentrations as follows (Nitrate-N shown as mg/Kg in sprayed/control subplots): Meadow 1, 11.8/3.5 (*P*<0.0001); Meadow 2, 9.2/3.9 (*P* = 0.0024); Hayfield 1, 6.1/1.3 (*P* = 0.0021); Hayfield 2, 6.8/4.2 (*P* = 0.167); Wasteland, 13.6/3.8 (*P*<0.0001). The nitrate levels in sprayed and control subplots in Hayfield 2 did not change. Other soil nutrients (P, K, Ca, Mg) were not affected by glyphosate application.

### Effect of Glyphosate on Bentgrass Survivorship, Vegetative Growth and Reproductive Potential

Introduced CB and RT plants had higher survivorship in subplots receiving glyphosate (56%) than control subplots (4%) (Table 2). This was confirmed by probit model analysis (*P*<0.001). RT and CB plants from sprayed subplots had higher final biomass than bentgrass in control subplots, and plot location did not affect final biomass (Table 2). The number of CB or RT tillers increased in sprayed subplots compared to controls (Fig. 3). The only exception was CB plants in the Wasteland plot where competition with invasive reed canarygrass negatively affected growth in all subplots (Fig. 3). CB plants had significantly more tillers than RT plants in sprayed subplots in Meadow 1; a similar but non-significant trend was observed in Meadow 2 and Hayfield 2.

Reproductive potential of CB and RT plants was measured in each year of the study. There was no significant plot effect (*P* = 0.524), so data were combined for analysis. As expected, CB and RT plants did not produce panicles or flowers in the first year (2008). In the second year (2009), RT plants in glyphosate subplots had an 86-fold increase in flower number compared to those in control subplots (Table 3). Thirteen RT plants produced a total of 238,896 flowers compared with 640 flowers on 3 RT control plants. Results in the third year (2010) were similar, although total flower numbers were probably reduced due to dry weather; 2009 had cooler temperatures and more rain during May and June which favored the cool-season grasses. RT seed collected in 2010 had 80% germination in the laboratory. Throughout the study, CB plants had a somewhat lower reproductive potential (flower number) than RT plants, but CB seeds had a relatively high germination rate of 74%.

## Discussion and Conclusion

### Effects of Glyphosate on Plant Community Composition and Soil Nitrate-N

Annual glyphosate application over a three-year period altered plant community composition in agricultural hayfields and natural meadows supporting our original hypothesis. This result is relevant to long-term risk assessment for HR crops that can escape cultivation because changes in plant community assemblages can produce negative impacts on other trophic levels [45]. Our results contrast with some previous reports that herbicides have little effect on plant species richness [20], [46], [47], [48], [18]. This discrepancy may be due, at least in part, to plant community types, differences in glyphosate application, and the metric (species richness) used to characterize changes in plant community composition over time. In our study, species richness declined in glyphosate-treated subplots in two plot locations (Meadow 2 and Hayfield 1). However, species richness data alone failed to capture the glyphosate-induced plant community changes in two other plots (Meadow 1 and Hayfield 2). This was because the number of new plant species appearing in the glyphosate-treated subplots was approximately equal to the number lost due to herbicide treatment. For example, the loss of some perennial plant species was often concurrent with an increase in weeds such as witchgrass (an annual warm-season C4 grass) or yellow rocket (a cool-season biennial), which were not present in subplots without glyphosate treatment. Furthermore, native perennial plants that declined in sprayed subplots included goldenrods such as tall goldenrod (*Solidago altissima* L.), rough goldenrod, giant goldenrod, and narrowleaf goldenrod (*Euthamia graminifolia* (L.) Nutt.), a trend that has been observed by other researchers [20]. Thus, measuring community composition over time is important for describing the response of complex agricultural or natural plant communities to annual glyphosate applications.

The only plot in our study that showed no effect from glyphosate on community composition was the Wasteland plot (Fig. 2). This plot was different because it had high soil P and K from past agricultural use and was dominated by one invasive plant species (reed canarygrass). This invasive regrew quickly after glyphosate application and competed with the introduced bentgrass plants, leading to very low survival rates. Nearly all of the introduced bentgrass plants died except for two RT plants in glyphosate-treated subplots. Based on our observations, it would be difficult for weedy bentgrass species to compete in this particular type of plant community regardless of the GR trait or glyphosate treatment.

The observation of increased nitrate-N in subplots sprayed with glyphosate was unexpected and difficult to explain, in part because the study did not include studies on microbial activity, soil nitrogen fluxes, and other minerals. A study in the Hubbard Brook ecosystem observed an increase in nitrate-N in a watershed where all vegetation was cut and suppressed for two years by herbicide application [49]. A study in young loblolly pine forests showed increased soil nitrogen in response to glyphosate, and the authors postulated the involvement of microbial mineralization [50], [51]. Increased soil nitrate-N was observed when turfgrass was removed with glyphosate, but nitrate-N levels returned to normal six weeks after reseeding [52]. Previous research has also suggested that the removal of living plants coupled with the deposition of dead plant material in the sprayed subplots could affect the nitrogen competition dynamics between microbes and plants [53]. In our plots, increased microbial mineralization of dead plant tissues in sprayed subplots may have lead to the increased nitrate-N observed in October-November. Alternatively, nitrate levels in our plots may have been affected by changes in the C:N balance because of shifts in the plant community and the amount or type of plant detritus in the sprayed subplots. In this study, high nitrate-N levels were dependant on glyphosate application and may have contributed to the growth of the introduced bentgrass plants because nitrogen is often the limiting element for plant growth [8]. However, the effect of nitrate-N on *Agrostis* growth and survivorship is difficult to separate from other glyphosate effects such as the removal of plant competitors. Further studies are needed to understand glyphosate effects on GR plant growth, competition and soil nutrient dynamics.

### Effect of Glyphosate on Bentgrass Growth and Reproductive Capacity

Our results supported the hypothesis that CB and RT plants with mimicked GR trait would have higher levels of survivorship, vegetative growth and reproductive potential with selection pressure. Transgenic GR bentgrasses could not used in this study for a variety of reasons including: 1) it would be necessary to obtain a Memorandum of Understanding (MOU) and approval from the company holding the intellectual property rights and patents for the plant product, 2) the experimental design required quantifying flower and seed number and this would have allowed transgene escape because bentgrasses were already present in the study sites, and 3) the GR trait has not been inserted into RT. Unintended transgene flow has been well documented in bentgrasses in Oregon [36]. Given our special attention to mimicking the GR trait, we feel confident that the results are applicable to transgenic bentgrasses in the habitats and plant communities studied.

CB and RT plants had more tillers, above-ground biomass, and flowers in sprayed subplots. The most likely explanation for this is that they experienced less competition from neighboring vegetation for resources such as light, soil nutrients, or soil moisture. This contrasts with the inference from some previous bentgrass studies [40], [41], but agrees with a study on ‘mock feral’ GR canola that showed increased crop fitness when exposed to glyphosate drift selection pressure [5] and a study that found glyphosate resistant *Brassica* species persisted as the dominant species after two annual applications of glyphosate drift [54].

CB and RT produced a high number of flowers and viable seeds in the second and third seasons of growth. RT plants in glyphosate-sprayed subplots had an 86-fold higher number of flowers compared to control subplots in 2009 (Table 3). CB plants in glyphosate-treated subplots also produced more flowers than controls. In addition, the germination rates for seed collected from CB and RT plants were above 70%. If the number of viable seeds is proportional to the number of flowers observed in 2009 and 2010, then seeds represent a significant pathway for dispersal of weedy bentgrasses carrying the GR trait. In addition, the large number of flowers suggests a strong potential for pollen-mediated transgene flow through either intraspecific or interspecific hybridization. Forced crosses and landscape-scale experiments have already demonstrated pollen-mediated gene flow between bentgrass species in the U.S. [38], [36], [39], [55]. In our study area, seven bentgrass species can be found in natural and cultural landscapes, and all of these species are able to hybridize [30].

The removal of vegetation by glyphosate is a disturbance that creates gaps in plant communities. Research has shown that, without gaps, seed recruitment in herbaceous plant communities (e.g. tallgrass prairie and pastures) is rare and episodic [56], but disturbed sites have a higher rate of seedling establishment [57]. A positive correlation between gap diameter, grass population growth rate, and panicle production has also been demonstrated [58]. These observations are consistent with our study where subplots with glyphosate disturbance had higher CB and RT survival and growth (Table 2). In addition, the gaps created by glyphosate were postively correlated with increased bentgrass panicle and tiller production. Thus, preventing or reducing gaps in plant communities could be one strategy for preventing the establishment of GR bentgrasses in natural and agricultural habitats in the Northeastern U.S.
